# 羧基化多壁碳纳米管改进的QuEChERS-气相色谱-串联质谱法同时检测蔬菜水果中34种农药及代谢物残留

**DOI:** 10.3724/SP.J.1123.2025.02001

**Published:** 2025-10-08

**Authors:** Xinzhong ZHANG, Xuemei WANG, Jun CHEN, Zhen ZHANG, Hui DING, Xinzhen DU, Xiaoquan LU

**Affiliations:** 1.高原交汇区水资源安全与水环境保护教育部重点实验室，西北师范大学化学化工学院，甘肃 兰州 730070; 1. Key Laboratory of Water Security and Water Environment Protection in Plateau Intersection，Ministry of Education，College of Chemistry and Chemical Engineering，Northwest Normal University，Lanzhou 730070，China; 2.兰州市食品药品检验检测研究院，甘肃 兰州 730050; 2. Lanzhou Institute for Food and Drug Control，Lanzhou 730050，China; 3.甘肃省生物电化学与环境分析重点实验室，甘肃 兰州 730070; 3. Key Laboratory of Bioelectrochemistry and Environmental Analysis of Gansu Province，Lanzhou 730070，China

**Keywords:** 羧基化多壁碳纳米管, QuEChERS, 气相色谱-三重四极杆质谱法, 蔬菜水果, 农药及代谢物, carboxylated multi-walled carbon nanotubes （MWCNTs-COOH）, QuEChERS, gas chromatography-triple quadrupole mass spectrometry （GC-MS/MS）, vegetables and fruits, pesticides and metabolites

## Abstract

为了有效监测农药在蔬菜水果中的残留水平，降低样品基质对目标物的干扰，本研究建立了羧基化多壁碳纳米管（MWCNTs-COOH）改进的QuEChERS联合GC-MS/MS技术对蔬菜水果中34种农药及代谢物残留的高通量检测方法。通过优化前处理过程及色谱、质谱分析条件，确定了蔬菜水果中34种农药及代谢物的最佳检测条件。具体方法如下：蔬菜水果样品经研磨粉碎，加入陶瓷均质子及QuEChERS EN-提取包，振荡离心后，取上清液转移至添加10 mg MWCNTs-COOH的净化管中，振荡离心后取2 mL上清液氮吹至近干，用含有内标的乙酸乙酯复溶，样品经0.22 μm尼龙微孔滤膜过滤后，经Agilent HP-5MS UI气相色谱柱（30 m×0.25 mm×0.25 μm）程序升温分离，GC-MS/MS多反应监测（MRM）模式检测，基质匹配内标法定量。在优化的实验条件下，34种农药及代谢物在相应的线性范围内呈良好的线性关系，相关系数均大于0.997 4，此方法具有较低的检出限（LOD：0.023～0.817 μg/kg）和定量限（LOQ：0.077～2.696 μg/kg）。在低、中、高3个加标水平下，34种农药及代谢物的加标回收率为78.9%～104.5%，相对标准偏差（RSD）为1.0%～7.8%。本方法净化效果显著，准确高效，适用于蔬菜水果中多组分农药及代谢物残留的检测。

## Abstract (en)

With the rapid advancement of industrialization， agricultural intensification， and urbanization， issues pertaining to environmental pollution and food safety have become increasingly prominent. The use of pesticides plays a vital role in augmenting agricultural yield and quality， thereby serving as a foundational element of modern agriculture. However， improper or excessive application can lead to pesticide residues， which pose a significant threat to ecological systems and human health. Therefore， it is essential to enhance the surveillance of pesticide residue levels and to pioneer novel， efficient analytical methodologies. Sample pretreatment constitutes a crucial stage in the detection pipeline. Consequently， the development of rapid， green， and efficient sample pretreatment techniques has advanced significantly and gained widespread application in recent years. To effectively monitor pesticide residues in fruits and vegetables while reducing matrix interference， we established a high-throughput method for detecting 34 pesticides and their metabolites. This method utilizes a modified QuEChERS approach with carboxylated multi-walled carbon nanotubes （MWCNTs-COOH） as a clean-up sorbent， followed by analysis with gas chromatography-tandem mass spectrometry （GC-MS/MS）. The detection conditions for 34 pesticide and metabolite residues in fruits and vegetables were established through the optimization of the pretreatment process， chromatographic conditions， and mass spectrometry parameters. Specifically， fruit and vegetable samples were homogenized and extracted using a commercial QuEChERS EN extraction kit. Following shaking and centrifugation， the supernatant was transferred to a purification tube containing 10 mg of MWCNTs-COOH. Subsequently， this supernatant was evaporated to near-dryness under a gentle stream of nitrogen， reconstituted in ethyl acetate containing an internal standard， and filtered through a 0.22 μm nylon membrane filter. The target substances were separated on a HP-5MS UI quartz capillary column （30 m×0.25 mm×0.25 μm） with a programmed temperature gradient， detected by GC-MS/MS in multiple reaction monitoring （MRM） mode， and quantified using a matrix-matched internal standard calibration method. A full scan was performed across an *m/z* range of 45-500. Retention times and characteristic fragment ions were identified using the NIST mass spectral library. The most intense fragment ion was selected as the precursor ion. Parameters including dwell time and collision energy were then optimized to select the optimal product ion for each transition. Acetonitrile was selected as the optimal extraction solvent， and 10 mg was determined to be the optimal dosage of MWCNTs-COOH for purification. Under the optimized conditions， all 34 pesticides and metabolites demonstrated good linearities within their respective concentration ranges， with correlation coefficients （*R*^2^） greater than 0.997 4. The method demonstrated low limits of detection （LODs） and quantification （LOQs）， ranging from 0.023 to 0.817 μg/kg and 0.077 to 2.696 μg/kg， respectively. At three spiked concentration levels （low， medium， and high）， the recoveries for the 34 pesticides and metabolites ranged from 78.9% to 104.5%， with relative standard deviations （RSDs） of 1.0% to 7.8%. Additionally， matrix effects were evaluated for six types of fruits and vegetables at a spiked mass concentration of 0.2 μg/mL. The results indicated significant differences in matrix effects among the sample matrices. Chives exhibited the strongest matrix effect， followed by chili peppers and celery. Bananas， grapes， and peaches were the least affected. Overall， strong matrix effects were observed in over 20% of the analyte-matrix combinations， with vegetables generally exhibiting a stronger influence than fruits. The evaluation confirmed significant differences in matrix effects for the target pesticides and metabolites across the various sample matrices. Consequently， a matrix-matched calibration method was employed for accurate quantification to correct for these effects. The developed method demonstrates high purification efficiency， accuracy， and reproducibility， rendering it highly suitable for the simultaneous determination of multiple pesticide and metabolite residues in fruits and vegetables.

蔬菜水果是我国居民膳食结构中重要的组成部分，富含人体必需的维生素、无机盐和膳食纤维，具有极高的营养价值^［1］^。蔬菜水果不宜贮存且受病虫害侵袭严重，必须使用各类农药进行防治。近年来，农药滥用问题日渐突出，特别是禁用农药的乱用及多种农药的复配使用^［2］^，使得农药及代谢物随着食物链在人体内蓄积，对人体健康造成极大威胁^［3］^。因此，必须加强对多农药残留的检测及长期监测。为加强农药残留风险管理，我国将相关标准统一合并后发布了《食品安全国家标准 食品中农药最大残留限量》（GB 2763-2012），并先后对该标准进行了5次修订，现行版本为2021年3月发布的《食品安全国家标准 食品中农药最大残留限量》（GB 2763-2021），该标准自2021年9月3日起正式实施，文中规定了2，4-D等564种农药在376种（类）食品中10 092项残留限量标准，其中蔬菜水果农药残留限量标准约占总数的56%。日本和韩国规定了食品中部分化学物质的农药残留最大限量，对于未列出的化学物质或列出化学物质但没有制定食品限量的化学物质，则采用“一律限量”，即 0.01 mg/kg。其他国家也相应制定了不同的限量标准。

农药残留检测技术作为保障食品安全的重要手段，必须具备准确性和安全性。目前，农药残留常用的检测方法主要有气相色谱法（GC）^［4，5］^、液相色谱法（HPLC）^［6，7］^、气相色谱-质谱法（GC-MS）和液相色谱-质谱法（HPLC-MS）^［8-10］^等。色谱法在单一农药或极性差异大的农药检测中发挥了重要作用，但随着检测农药种类的增加，色谱-质谱法灵敏度高、定性定量准确的优势更加明显。由于蔬菜水果中富含色素、脂肪、纤维素等杂质，对色谱信号峰易造成明显干扰，容易产生基质效应，因此，选择合适的前处理技术至关重要。目前，农药残留的前处理方法主要包括固相萃取法^［11］^、液液萃取法^［12，13］^、QuEChERS（quick、easy、cheap、effective、rugged、safe）法^［14］^等。传统的萃取方法需要较多的有机溶剂，且操作复杂，耗时长，相比之下，QuEChERS法作为一种快速、简单、廉价、有效、耐用和安全的方法，消耗的溶剂较少，且萃取效率更高，已成为广泛使用的农药残留前处理技术^［1，15］^。与此同时，还具备配方可调整的灵活性，常用的净化材料主要有十八烷基键合硅胶（C_18_）、石墨化炭黑（GCB）、乙二胺-*N*-丙基硅烷（PSA）和碳纳米管等（CNTs）^［16］^。

CNTs是具有特殊结构的碳材料，比表面积大，吸附能力优越，化学性质稳定，与传统的GCB相比，具备更强的杂质净化能力，且对农药及代谢物吸附能力低^［17，18］^。陈婷等^［16］^ 以GMWCNTs作为净化吸附剂， 对肉桂、葛根等药食同源食品进行净化，有效去除了样品杂质；潘永波等^［19］^利用NH_2_-MWCNTs对热带水果进行净化，农药回收率均大于60%，性能良好。MWCNTs-COOH是在原始MWCNTs的基础上，通过强酸氧化等方法引入了羧基（-COOH）官能团，从而改善了其在水中或其他溶剂中的溶解性和分散性，加强了反应活性，是一种新型净化材料^［20］^。本研究通过优化MWCNTs-COOH用量，建立了MWCNTs-COOH改进的QuEChERS联合气相色谱-串联质谱法（GC-MS/MS）测定蔬菜水果中34种农药及代谢物（共分为6类：有机磷农药、有机氯农药、拟除虫菊酯类农药、二硝基甲苯胺类除草剂、二甲酰亚胺类杀菌剂、杂环类杀菌剂）残留的方法，考察了不同样品中农药及代谢物的基质效应，以期为农药多残留检测和食品安全监管提供技术支持。

## 1 实验部分

### 1.1 仪器、试剂与材料

Agilent 7890B+7000D三重四极杆气相色谱-质谱仪（美国Agilent公司）；Centrifuge 5810R高速离心机（德国 Eppendorf公司）；VXR涡旋振荡器（德国IKA公司）；Milli-Q超纯水机（美国Millipore公司）。

34种农药及代谢物标准品和1种内标标准品（质量浓度均为100 μg/mL，天津阿尔塔科技有限公司）。甲醇、乙腈、乙酸乙酯、丙酮、甲酸（色谱纯，德国Merck公司），MWCNTs-COOH（纯度>98%，中国科学院成都有机化学有限公司），陶瓷均质子（北京科德诺思技术有限公司），QuEChERS提取包（美国Agilent公司），QuEChERS净化管（天津博纳艾杰尔科技有限公司）。实际样品均购自本地超市。

### 1.2 标准溶液的配制

分别准确吸取100 μL 34种农药及代谢物标准品于 10 mL 容量瓶中，用乙酸乙酯稀释、定容并摇匀，配制成质量浓度为1 μg/mL的标准储备液，于-20 ℃下冷冻保存。用空白基质提取液稀释标准储备液，配制成所需质量浓度的基质匹配混合标准溶液，进样测定。

### 1.3 实验方法

#### 1.3.1 试样制备

取500 g样品可食部分，用粉碎机充分搅碎、混匀，得到试验所需样品，于-18 ℃下冷冻保存。

#### 1.3.2 样品前处理

将样品解冻至室温，精密称取10.00 g（±0.05 g）置于50 mL塑料离心管中，准确加入10 mL乙腈、QuEChERS-EN提取包（4.00 g硫酸镁、1.00 g氯化钠、1.00 g柠檬酸钠、0.50 g柠檬酸氢二钠）及1颗陶瓷均质子，剧烈振荡1 min，4 200 r/min离心5 min。吸取6 mL上清液加入到15 mL净化管（含0.9 g硫酸镁、0.15 g PSA、0.01 g MWCNTs-COOH）中，涡旋混匀1 min。4 200 r/min离心5 min，准确吸取2.00 mL上清液于10 mL试管中，40 ℃水浴中氮气吹至近干，加入2.00 mL含内标（环氧七氯：200.0 ng/mL）的乙酸乙酯复溶，通过0.22 μm尼龙微孔滤膜，用于测定。

### 1.4 仪器条件

#### 1.4.1 色谱条件

HP-5MS UI气相色谱柱（30 m×0.25 mm×0.25 μm，美国Agilent公司）采用程序升温模式进行样品分析（表1）。进样口温度：280 ℃；载气：氦气（99.999%）；进样体积：1 μL；流速：1 mL/min。分流模式：不分流进样。

**表1:** GC升温程序

Item	Rate**/** **（**℃/min）	Temperature**/**℃	Hold time/min	Run time/min
Initial value		60	1	1
Gradient 1	40	170	0	3.75
Gradient 2	10	310	3.3	21.05

#### 1.4.2 质谱条件

离子源类型：电子轰击电离（EI）源；离子源温度：230 ℃；扫描方式：二级质谱多反应监测（MRM）分段扫描，第1段3.35～8.80 min，第2段8.80～13.00 min，第3段13.00～21.05 min；电子能量模式：使用调谐设置；电子能量：70 eV；传输线温度：280 ℃；溶剂延迟：3.5 min。农药及代谢物的质谱参数详见表2。

**表2:** 34种农药及代谢物和内标物质的保留时间和质谱参数

No.	Compound	Retention time/min	Monitoring ion pairs （*m/z*）	Dwell times/ms	Collision energies/eV
1	dichlorvos^1）^	4.450	184.9>93.0^*^， 108.9>79.0	15，10	15，15
2	methamidophos^1）^	4.489	141.0>95.0^*^， 141.0>79.0	10，10	5，10
3	acephate^1）^	5.438	136.0>94.0^*^， 136.0>42.0	10，10	5，15
4	omethoate^1）^	6.462	155.9>110.0^*^， 109.9>79.0	15，10	5，15
5	phorate^1）^	7.107	260.0>75.0^*^， 230.9>128.9	15，15	10，10
6	*α*-HCH^2）^	7.239	216.9>181.0^*^， 181.1>145.0	10，10	5，15
7	dimethoate^1）^	7.423	92.9>63.0^*^， 86.9>46.0	10，10	10，15
8	*β*-HCH^2）^	7.650	216.9>181.0^*^， 181.0>145.0	10，10	5，15
9	*γ*-HCH^2）^	7.725	217.0>181.1^*^， 180.9>145.0	10，10	5，15
10	isazophos^1）^	8.094	161.0>146.0^*^， 161.0>119.0	15，15	5，5
11	*δ*-HCH^2）^	8.112	217.0>181.1^*^， 181.1>145.1	10，10	5，15
12	chlorothalonil^2）^	8.196	263.8>229.0^*^， 263.8>168.0	15，15	20，25
13	*o，p′*-dicofol^2）^	9.076	139.0>111.0^*^， 250.9>138.9	10，10	15，15
14	phorate-sulfoxide^1）^	9.410	199.0>142.9^*^， 96.9>64.9	15，15	10，20
15	phorate-sulfone^1）^	9.411	153.0>97.0^*^， 124.9>96.9	10，10	10，10
16	fenthion^1）^	9.452	278.0>169.0^*^， 278.0>109.0	10，10	15，15
17	chlorpyrifos^1）^	9.482	198.9>171.0^*^， 196.9>169.0	5，5	15，15
18	*p，p′*-dicofol^2）^	9.571	139.0>111.0^*^， 250.9>138.9	10，10	8，8
19	isocarbophos^1）^	9.610	135.9>108.0^*^， 135.9>69.0	5，5	15，15
20	isofenphos-methyl^1）^	9.921	241.1>199.1^*^， 199.0>121.0	10，10	15，15
21	pendimethalin^4）^	10.035	251.8>162.2^*^， 251.8>161.1	10，10	10，15
22	heptachlor epoxide^#^	10.105	352.8>262.9^*^， 354.8>264.9	10，10	15，15
23	procymidone^5）^	10.352	96.0>67.1^*^， 96.0>53.1	5，5	15，10
24	methidathion^1）^	10.510	144.9>58.1^*^， 144.9>85.0	10，10	15，5
25	profenofos^1）^	11.036	338.8>268.7^*^， 207.9>63.0	10，10	15，30
26	dieldrin^2）^	11.189	262.9>193.0^*^， 262.9>191.0	10，10	35，35
27	fenthion-sulfoxide^1）^	11.787	278.0>169.0^*^， 278.0>109.0	10，10	15，15
28	fenthion-sulfone^1）^	11.869	309.9>105.0^*^， 135.9>92.0	10，10	10，10
29	triazophos^1）^	12.152	161.2>106.1^*^， 161.2>134.2	5，5	5，10
30	bifenthrin^3）^	13.392	181.2>166.2^*^， 181.2>165.2	25，25	25，10
31	cyhalothrin^3）^	14.303	197.0>161.0^*^， 197.0>141.0	25，25	20，20
32	cypermethrin^3）^	16.078	163.0>127.0^*^， 163.0>91.0	25，25	10，15
33	fenvalerate^3）^	16.977	224.9>119.0^*^， 167.0>125.1	25，25	15，15
34	difenoconazole^6）^	17.332	322.8>264.8^*^， 264.9>202.0	25，25	15，20
35	deltamethrin^3）^	17.563	252.9>93.0^*^， 250.7>172.0	25，25	15，5

* quantitative ion； # internal standard； 1） organophosphorus pesticides； 2） organochlorine pesticides； 3） pyrethroid pesticides； 4） dinitrotoluene herbicides； 5） dicarboximide herbicides； 6） heterocyclic fungicides.

## 2 结果与讨论

### 2.1 质谱条件优化

根据MRM的多反应监测模式，设定多时间段和扫描通道，在*m/z* 45~500范围内全扫描，利用NIST标准库匹配，以确定保留时间和一级碎片离子，选择离子强度高的一级碎片离子为母离子，通过优化驻留时间、碰撞能量等参数选择最优的定量离子^［21，22］^。农药及代谢物和内标物质的总离子流图见图1。

**图1:**
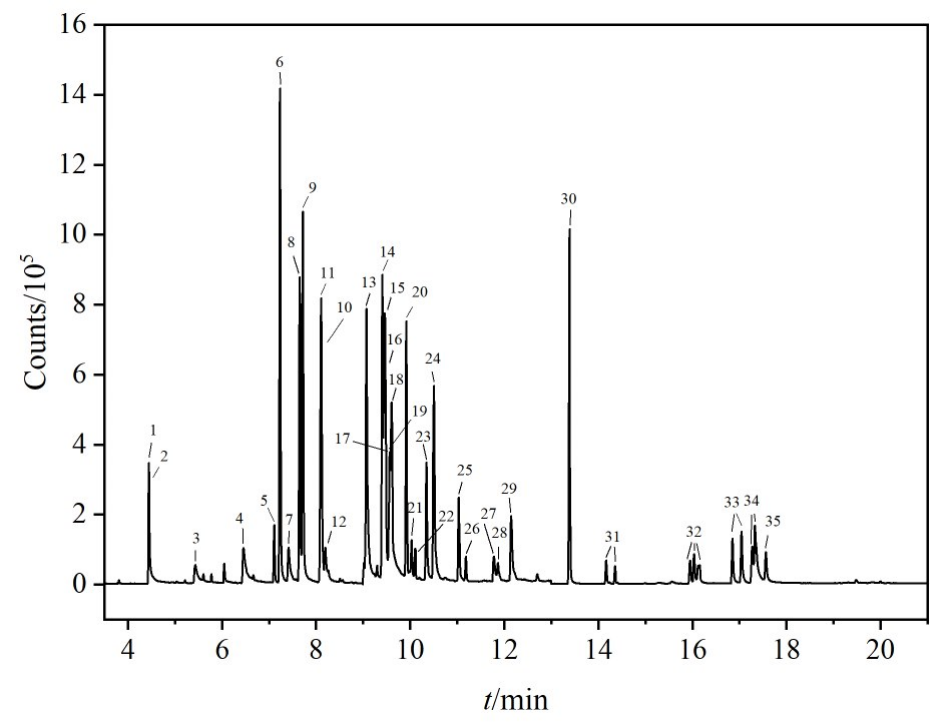
34种农药及代谢物和内标物质的总离子流图 For peak Nos.， see Table 2.

### 2.2 提取溶剂优化

溶剂的选择对目标物的提取具有重要影响，选择甲醇、乙腈、乙酸乙酯、丙酮、正己烷5种有机溶剂作为提取溶剂，考察对34种农药及代谢物的提取效果（见图2）。根据有机溶剂极性顺序表^［23］^，查得5种溶剂的极性大小依次为甲醇（6.6）>乙腈（6.2）>丙酮（5.4）>乙酸乙酯（4.3）>正己烷（0）。由于提取盐包中含有1 g柠檬酸钠和0.5 g柠檬酸氢二钠，因此不再额外添加酸性物质。实验结果表明，5种溶剂对于农药及代谢物的平均回收率分别为38.3%、92.3%、78.4%、72.4%和44.1%，因此选择乙腈作为提取溶剂。

**图2:**
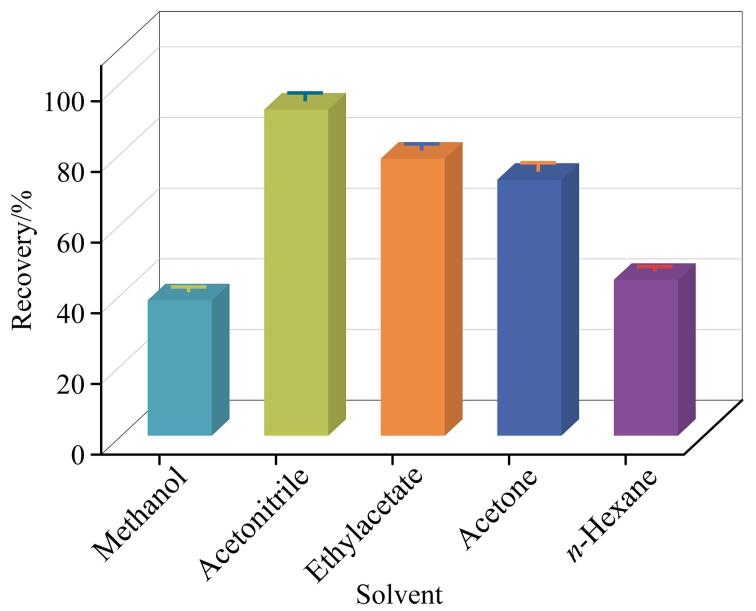
采用5种提取溶剂时34种农药及代谢物的平均回收率（*n*=3）

### 2.3 净化条件优化

MWCNTs-COOH中引入-COOH改善了其在水中或其他溶剂中的溶解性和分散性，增加了活性位点。在实际样品测定中，添加MWCNTs-COOH后的净化能力优于GCB，有机磷类农药、拟除虫菊酯农药及其他农药的平均回收率分别提高了5.3%、4.2%和2.1%。因此，主要优化了MWCNTs-COOH的添加量。结果如图3a所示，随着MWCNTs-COOH的增加，净化液的颜色逐渐变浅，增至10 mg 后未见明显变化，表明MWCNTs-COOH对样品中的杂质具有较好的净化能力。如图3b所示，MWCNTs-COOH的添加量为10.00 mg时，平均回收率为94.1%，进一步增加时，平均回收率未见明显变化，甚至出现下降趋势。这是由于MWCNTs-COOH上富含活性位点，与杂质吸附完全后，可能与样品中的目标物发生作用，降低平均回收率。

**图3:**
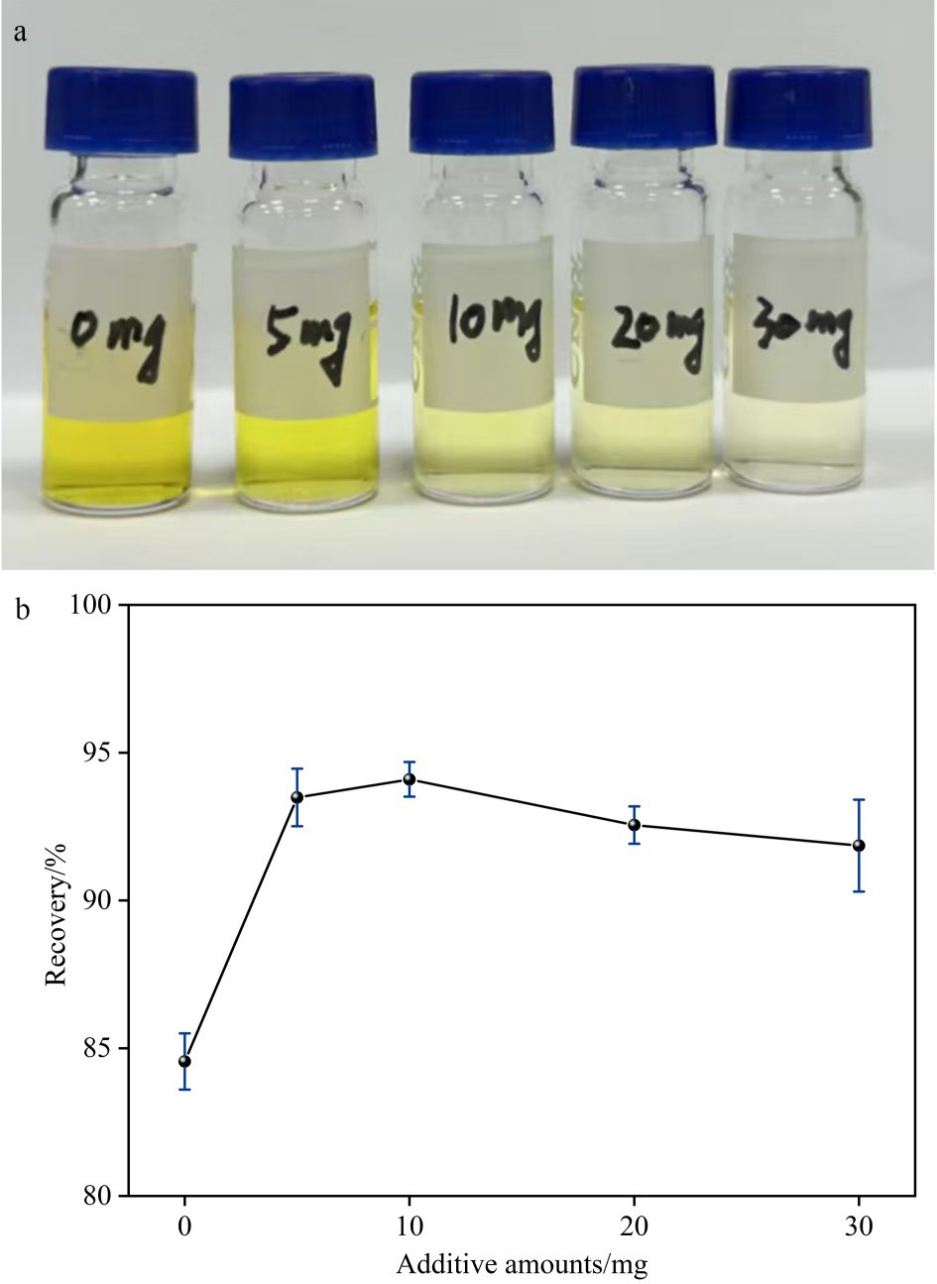
添加不同量MWCNTs-COOH时（a）溶剂的颜色和（b）34种农药及代谢物的平均回收率（*n*=3）

### 2.4 方法学验证

#### 2.4.1 线性范围、相关系数、检出限和定量限

取芹菜样品，按1.3节方法进行试样制备和样品前处理，得到空白基质提取液，再按照1.2节方法配制系列质量浓度的基质匹配混合标准溶液，按1.4节条件进样分析。以目标化合物与内标物的峰面积比为纵坐标（*y*），目标化合物与内标物的质量浓度比为横坐标（*x*，ng/mL），绘制基质匹配标准曲线。实验数据表明，34种农药在各自范围内线性关系良好，相关系数（*R* ^2^）均≥0.997 4。分别以*S/N*=3和*S/N*=10作为方法检出限（LOD）和定量限（LOQ），结果表明，LOD为0.023~0.817 μg/kg，LOQ为0.077~2.696 μg/kg，相关数据结果见表3。上述实验结果表明，本方法具有较高的灵敏度。

**表3:** 线性范围、线性方程、相关系数、检出限、定量限、加标回收率及标准偏差（*n*=6）

Analyte	Linear range/ （μg/kg）	Linear equation	*R* ^2^	Recoveries （RSDs）/%			LOD/ （μg/kg）	LOQ/ （μg/kg）
				5 μg/kg	20 μg/kg	50 μg/kg		
Dichlorvos	1.363-500	*y*=2.905*x*+0.024	0.9998	92.2 （3.6）	93.4 （4.1）	95.3 （3.2）	0.496	1.363
Methamidophos	2.482-500	*y*=2.163*x*+0.045	0.9998	91.6 （3.5）	92.5 （4.7）	93.6 （3.3）	0.752	2.482
Acephate	1.891-500	*y*=1.935*x*+0.032	0.9998	79.5 （4.2）	83.5 （5.2）	87.9 （4.2）	0.573	1.891
Omethoate	0.943-500	*y*=3.591*x*+0.074	0.9980	93.5 （3.4）	92.3 （5.2）	93.5 （4.4）	0.286	0.943
Phorate	0.265-500	*y*=2.420*x*-0.010	0.9999	99.8 （4.2）	98.4 （5.3）	103.9 （4.2）	0.080	0.265
*α*-HCH	0.899-500	*y*=7.664*x*-0.030	0.9996	92.4 （4.2）	91.8 （5.4）	91.6 （2.0）	0.272	0.899
Dimethoate	0.976-500	*y*=2.381*x*+0.024	0.9995	95.3 （4.8）	94.3 （3.2）	94.6 （2.9）	0.296	0.976
*β*-HCH	1.384-500	*y*=5.032*x*+0.012	0.9999	88.4 （1.7）	90.9 （4.9）	92.7 （6.1）	0.419	1.384
*γ*-HCH	1.666-500	*y*=5.143*x*-0.027	0.9995	93.8 （2.9）	94.2 （6.6）	93.5 （3.4）	0.505	1.666
Isazophos	1.098-500	*y*=4.834*x*+0.015	0.9999	92.9 （3.2）	91.4 （3.9）	92.4 （4.4）	0.333	1.098
*δ*-HCH	2.439-500	*y*=3.744*x*+0.021	0.9998	90.8 （3.2）	88.2 （2.4）	86.8 （3.7）	0.739	2.439
Chlorothalonil	0.950-500	*y*=1.719*x*-0.013	0.9999	78.9 （3.3）	83.6 （4.2）	88.5 （5.1）	0.288	0.950
*o，p′*-Dicofol	0.271-500	*y*=21.040*x*+0.195	0.9994	82.3 （1.3）	81.5 （3.2）	85.9 （4.5）	0.082	0.271
Phorate-sulfoxide	0.509-500	*y*=3.387*x*+0.016	0.9996	92.5 （5.1）	94.3 （4.9）	95.4 （4.8）	0.154	0.509
Phorate-sulfone	1.169-500	*y*=8.867*x*+0.091	0.9991	94.3 （4.9）	95.3 （4.2）	95.9 （2.3）	0.354	1.169
Fenthion	0.180-500	*y*=11.380*x*+0.138	0.9997	97.7 （4.1）	98.6 （3.1）	101.3 （2.0）	0.054	0.180
Chlorpyrifos	0.900-500	*y*=4.398*x*+0.009	0.9999	94.3 （5.2）	95.2 （2.5）	93.4 （7.8）	0.273	0.900
*p，p′*-Dicofol	0.588-500	*y*=14.580*x*-0.021	0.9997	85.9 （2.6）	82.1 （4.1）	90.4 （4.7）	0.178	0.588
Isocarbophos	1.586-500	*y*=7.738*x*+0.049	0.9997	89.8 （5.3）	93.5 （3.6）	95.4 （1.9）	0.481	1.586
Isofenphos-methyl	0.194-500	*y*=12.630*x*+0.061	0.9999	97.6 （6.3）	95.9 （5.0）	99.9 （4.7）	0.059	0.194
Pendimethalin	1.045-500	*y*=1.178*x*-0.016	0.9994	94.1 （4.9）	89.4 （3.7）	92.3 （4.7）	0.317	1.045
Procymidone	2.021-500	*y*=5.484*x*+0.746	0.9998	92.5 （6.1）	94.3 （1.9）	89.9 （4.2）	0.612	2.021
Methidathion	0.158-500	*y*=11.540*x*-0.199	0.9981	93.4 （2.9）	94.7 （3.8）	96.4 （3.0）	0.048	0.158
Profenofos	0.844-500	*y*=1.735*x*+0.011	0.9997	92.4 （5.0）	88.3 （3.7）	93.2 （3.8）	0.256	0.844
Dieldrin	2.696-500	*y*=0.838*x*+0.005	0.9998	89.9 （6.2）	90.4 （4.8）	91.4 （3.6）	0.817	2.696
Fenthion-sulfoxide	2.017-500	*y*=1.864*x*+0.014	0.9992	90.3 （3.7）	92.2 （3.5）	90.5 （5.2）	0.611	2.017
Fenthion-sulfone	1.403-500	*y*=1.025*x*+0.011	0.9997	93.2 （2.1）	91.3 （4.8）	88.5 （3.2）	0.425	1.403
Triazophos	1.395-500	*y*=2.773*x*+0.033	0.9996	90.4 （3.4）	95.4 （1.0）	94.3 （1.1）	0.423	1.395
Bifenthrin	0.077-500	*y*=12.250*x*+0.042	0.9999	98.8 （1.6）	97.4 （2.5）	104.5（4.9）	0.023	0.077
Cyhalothrin	1.848-500	*y*=2.591*x*+0.013	0.9999	94.6 （3.8）	95.2 （4.1）	93.4 （2.7）	0.560	1.848
Cypermethrin	1.478-500	*y*=3.947*x*+0.089	0.9998	92.5 （4.3）	91.5 （3.2）	92.5 （3.7）	0.448	1.478
Fenvalerate	1.519-500	*y*=5.674*x*-0.031	0.9991	94.1 （3.8）	94.7 （3.9）	95.3 （2.5）	0.460	1.519
Difenoconazole	1.135-500	*y*=6.678*x*+0.029	0.9992	94.3 （2.1）	95.9 （4.8）	94.3 （5.3）	0.344	1.135
Deltamethrin	1.996-500	*y*=1.530*x*-0.037	0.9974	90.4 （4.3）	94.3 （4.7）	95.1 （4.9）	0.605	1.996

*y：* peak area ratio of target compound to internal standard； *x*： mass concentration ratio of target compound to internal standard.

#### 2.4.2 回收率和精密度

在空白样品基质中分别添加低、中、高3个水平（5、20、50 μg/kg）的混合标准工作溶液，进行加标回收试验，每个加标水平制备6个平行样，计算回收率和相对标准偏差（RSD），实验结果见表3。结果显示，在3个加标水平下，34种农药及代谢物的加标回收率为78.9%~104.5%，RSD为1.0%~7.8%。上述结果说明本方法的回收率和精密度均较高，通用性强，能够满足蔬菜水果中 34种农药及代谢物的快速筛查与确证要求。

### 2.5 基质效应

基质效应（matrix effect，ME）是指样品中除分析物以外的组分对分析结果的影响。这些组分可能会干扰分析物的测定，导致分析结果不准确，在多组分农药的分析中不可避免^［24］^。基质效应会导致目标化合物响应信号的增强或抑制^［25］^。本文采用相对响应值法来评价ME，即ME=*B*/*A* ×100% ，式中：*A*为纯溶剂中目标物的响应值，*B*为样品基质中添加相同浓度目标物的响应值。当ME<80%时，为基质抑制效应；当80%≤ME≤120%时，为弱基质效应，表明基质对目标物的影响较弱，可忽略不计；当ME≥120%时，为基质增强效应。当ME介于50%~80%和120%~150%时为中等基质效应；当ME<50%或ME>150%时为强基质效应^［26］^。

在GC-MS分析中，基质效应普遍存在，且多为基质增强效应。样品中的不易挥发化合物易在进样口和衬管处富集沉积，影响结果的准确性和灵敏度。本研究考察了韭菜、辣椒、芹菜、香蕉、葡萄、桃子6种蔬菜水果在0.2 μg/mL水平下的基质效应（见图4a）。结果表明：不同样品对于目标物的基质效应差异显著，韭菜基质效应最强，辣椒次之，芹菜、香蕉、葡萄、桃子受基质效应影响较低。由图4b可知，蔬菜水果中的强基质效应占比超过20%，蔬菜的基质效应影响比水果的基质效应影响更大，为了降低此影响，采用基质匹配标准曲线法进行定量校正。

**图4:**
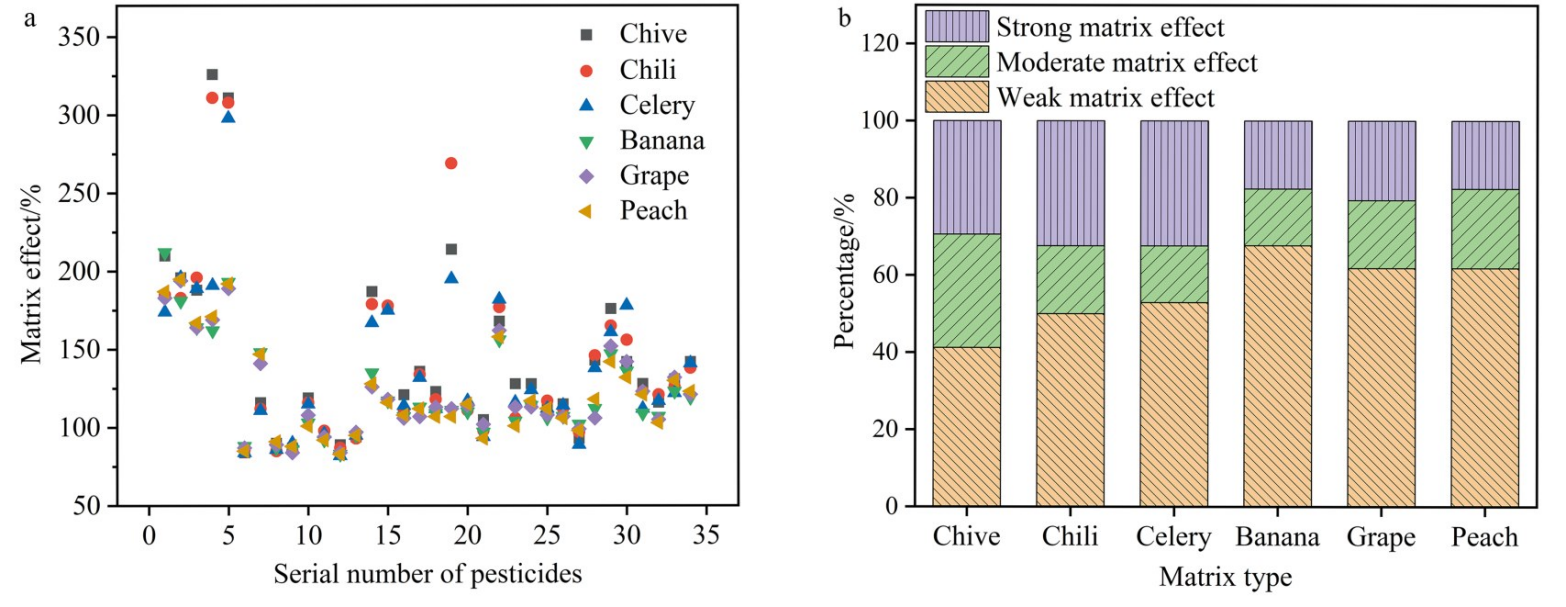
（a）6种实际样品中农药及代谢物在0.2 μg/mL水平下的基质效应和（b）34种农药及代谢物的基质效应分布及占比

### 2.6 实际样品分析

利用所建方法对14 种市售蔬菜水果（韭菜、辣椒、菠菜、白菜、黄瓜、生姜、番茄、茄子、豇豆、苹果、梨、香蕉、葡萄、柠檬）进行检测，1 000批次蔬菜水果中农药及代谢物的检出率为91.6%，检出一种农药的占比为24.5%，检出两种农药及代谢物的占比为33.4%，检出3种及以上的占比为42.1%（见图5a），农药复配使用现象较为明显。

**图5:**
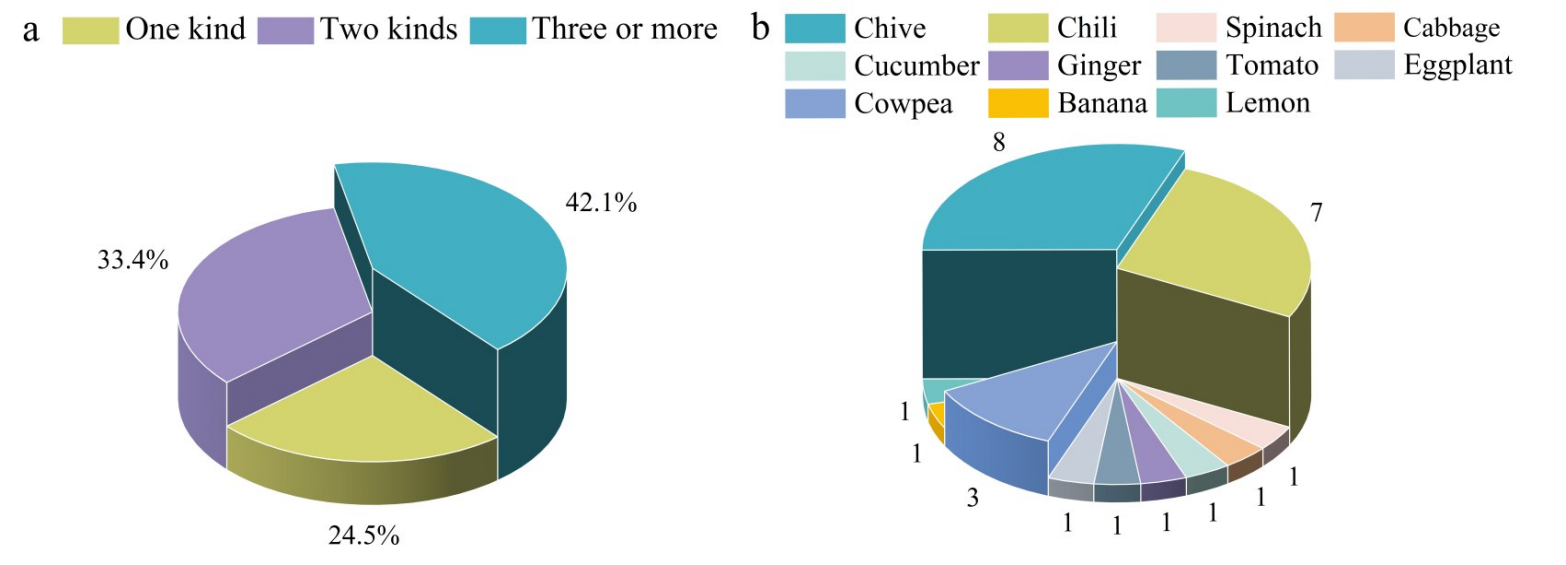
（a）农药检出数量占比和（b）不合格样品种类及数量

根据GB 2763-2021要求，检出不合格蔬菜水果26批（苹果、梨、葡萄均合格）（见图5b），不合格率为2.6%，不合格项目为毒死蜱（21批，含量为0.083～0.66 mg/kg）、水胺硫磷（1批，含量为0.82 mg/kg）、联苯菊酯（2批，含量为0.088～0.44 mg/kg）、氯氟氰菊酯（1批，含量为2.41 mg/kg）和氯氰菊酯（1批，含量为0.59 mg/kg）。

## 3 结论

本文通过优化提取溶剂和MWCNTs-COOH的添加量，建立了MWCNTs-COOH改进的QuEChERS-GC-MS/MS高通量检测蔬菜水果中34种农药及代谢物的方法。该方法具有操作简便、稳定可靠等优势。通过对6种蔬菜水果中的基质效应进行考察，发现基质效应在不同样品中差异显著，中等基质效应和强基质效应占比较高，因此，需采用基质匹配标准曲线内标法定量校正，可极大降低样品基质带来的干扰。本研究建立的方法有助于为蔬菜水果中多组分农药及代谢物检测提供新的思路，为市场监管提供技术支持。

## References

[1] Tankiewicz M， Berg A . Microchem J， 2022， 181： 107794

[2] Xie S E， Feng Y Y， Li Z， et al . Chinese Journal of Analysis Laboratory， 2025， 44（8）： 1139

[3] Tsagkaris A S， Uttl L， Pulkrabova J， et al . Appl Sci， 2020， 10： 565

[4] Merib J， Simão V， Dias A N， et al . J Chromatogr A， 2013， 1321： 30 24239037 10.1016/j.chroma.2013.10.080

[5] Fang Y Y， Zhou F Z， Zhang Q， et al . Talanta， 2023， 267： 125223 37748274 10.1016/j.talanta.2023.125223

[6] Zhao J L， Huang P F， Wang X M， et al . Sep Purif Technol， 2022， 287： 120608

[7] Watanabe E， Kobara Y， Baba K， et al . Food Chem， 2014， 154： 7 24518309 10.1016/j.foodchem.2013.12.075

[8] Damale R D， Dutta A， Shaikh N， et al . Food Chem， 2023， 407： 135179 36521392 10.1016/j.foodchem.2022.135179

[9] Gong X Y， Xu L Y， Huang S M， et al . Anal Chim Acta， 2022， 1218： 339982 35701037 10.1016/j.aca.2022.339982

[10] Harischandra N R， Pallavi M S， Bheemanna M， et al . Food Chem， 2021， 347： 128986 33515969 10.1016/j.foodchem.2020.128986

[11] Li W， Zhang Y， Jia H， et al . Microchem J， 2019， 150： 104168

[12] Srivastava A， Singh M， Singh S， et al . Measurement， Analysis and Remediation of Environmental Pollutants， 1st ed. Berlin： Springer Press， 2020

[13] Miyardan F N， Afshar Mogaddam M R， Farajzadeh M A， et al . Microchem J， 2022， 182： 107884

[14] Rutkowska E， Łozowicka B， Kaczyński P . J Chromatogr A， 2020， 1614： 460738 31806271 10.1016/j.chroma.2019.460738

[15] Buah-Kwofie A， Humphries M S . J Chromatogr B， 2019， 1105： 85 10.1016/j.jchromb.2018.12.010 30576889

[16] Chen T， Yan J， Zhang W， et al . The Food Industry， 2021， 42（6）： 489

[17] Yuan L J， Zhang K X， Li Z， et al . Food and Fermentation Industries， 2025， 50（2）： 326

[18] Yi C S， Liu R， Wu Z P， Chinese Journal of Chromatography ， 2024， 42（3）： 282 10.3724/SP.J.1123.2023.07018 PMC10951808 38503705

[19] Pan Y B， Zhang M Y， Wan N， et al . Chinese Journal of Pesticide Science， 2024， 26（1）： 189

[20] Yang J， Zhang X， Wang X， et al . J Chromatogr A， 2022， 1681： 463459 36108351 10.1016/j.chroma.2022.463459

[21] Zhou J， Liu G， Guo Z， et al . Chem Eng J， 2023， 455： 140167

[22] Che S， Peng X， Zhuge Y， et al . J Agric Food Chem， 2022， 70（49）： 15390 36417496 10.1021/acs.jafc.2c05980

[23] Yu D R， Li R D . Hubei Chemical Industry， 1984， 1（2）： 65

[24] Zhang X N， Zhou Y， Huang X Y， et al . Food Chem， 2023， 407： 135115 36508865 10.1016/j.foodchem.2022.135115

[25] Shivankar B R， Singh C P， Krishnamurty S . Appl Surf Sci， 2023， 619： 156745

[26] Li X Y， Zhao B L， Luo L J， et al . TrAC-Trends Anal Chem， 2023， 158： 116901

